# Evolution of female promiscuity in Passerides songbirds

**DOI:** 10.1186/s12862-019-1493-1

**Published:** 2019-08-14

**Authors:** Jan T. Lifjeld, Jostein Gohli, Tomáš Albrecht, Eduardo Garcia-del-Rey, Lars Erik Johannessen, Oddmund Kleven, Petter Z. Marki, Taiwo C. Omotoriogun, Melissah Rowe, Arild Johnsen

**Affiliations:** 1 Natural History Museum, University of Oslo, P.O. Box 1172, Blindern, NO-0318 Oslo, Norway; 2 Institute of Vertebrate Biology, Academy of Sciences of the Czech Republic, v.v.i., Květná 8, CZ-67502 Brno, Czech Republic; 3 Department of Zoology, Charles University in Prague, Viničná 7, CZ-12844 Prague, Czech Republic; 4 Macaronesian Institute of Field Ornithology, C/ Elias Ramos Gonzalez 5, 3-H, 38001 Santa Cruz de Tenerife, Canary Islands Spain; 5 Norwegian Institute for Nature Research, P.O. Box 5685, Torgarden, NO-7485 Trondheim, Norway; 6 Center for Macroecology, Evolution and Climate, Natural History Museum of Denmark, University of Copenhagen, Universitetsparken 15, DK-2100 Copenhagen, Denmark; 7 A.P. Leventis Ornithological Research Institute, University of Jos, Jos, Nigeria; 8 Biotechnology Unit, Department of Biological Sciences, Elizade University, P.M.B. 002, Ilara-Mokin, Ondo State Nigeria; 9 Centre for Ecological and Evolutionary Synthesis, Department of Biosciences, University of Oslo, P.O. Box 1066, Blindern, NO-0316 Oslo, Norway

**Keywords:** Extrapair paternity, Life history, Mating system, Pair bond, Parental care, Sexual selection

## Abstract

**Background:**

Female promiscuity is highly variable among birds, and particularly among songbirds. Comparative work has identified several patterns of covariation with social, sexual, ecological and life history traits. However, it is unclear whether these patterns reflect causes or consequences of female promiscuity, or if they are byproducts of some unknown evolutionary drivers. Moreover, factors that explain promiscuity at the deep nodes in the phylogenetic tree may be different from those important at the tips, i.e. among closely related species. Here we examine the relationships between female promiscuity and a broad set of predictor variables in a comprehensive data set (*N* = 202 species) of Passerides songbirds, which is a highly diversified infraorder of the Passeriformes exhibiting significant variation in female promiscuity.

**Results:**

Female promiscuity was highly variable in all major clades of the Passerides phylogeny and also among closely related species. We found several significant associations with female promiscuity, albeit with fairly small effect sizes (all R^2^ ≤ 0.08). More promiscuous species had: 1) less male parental care, particularly during the early stages of the nesting cycle (nest building and incubation), 2) more short-term pair bonds, 3) greater degree of sexual dichromatism, primarily because females were drabber, 4) more migratory behaviour, and 5) stronger pre-mating sexual selection. In a multivariate model, however, the effect of sexual selection disappeared, while the other four variables showed additive effects and together explained about 16% of the total variance in female promiscuity. Female promiscuity showed no relationship with body size, life history variation, latitude or cooperative breeding.

**Conclusions:**

We found that multiple traits were associated with female promiscuity, but these associations were generally weak. Some traits, such as reduced parental care in males and more cryptic plumage in females, might even be responses to, rather than causes of, variation in female promiscuity. Hence, the high variation in female promiscuity among Passerides species remains enigmatic. Female promiscuity seems to be a rapidly evolving trait that often diverges between species with similar ecologies and breeding systems. A future challenge is therefore to understand what drives within-lineage variation in female promiscuity over microevolutionary time scales.

**Electronic supplementary material:**

The online version of this article (10.1186/s12862-019-1493-1) contains supplementary material, which is available to authorized users.

## Background

More than three decades of molecular paternity studies in hundreds of bird species have revealed a fascinating variation in the extent to which females engage in multiple mating and produce a clutch of eggs with multiple sires [1–3]. This behaviour is often referred to as “extrapair copulation”, but here we term it “promiscuity” as a more general concept that also encompasses social polyandry and species without pair bonds. The reasons why female promiscuity is so variable among species are not well understood [4, 5], nor is it clear whether female promiscuity is adaptive for females [4, 6]. Hence, explaining the ecology and evolution of female promiscuity in birds, as well as in other taxa, is still a major challenge in evolutionary biology.

Several hypotheses explaining female promiscuity variation have been proposed and tested in various comparative and meta-analytical approaches [1, 2, 7–9]. The first set of hypotheses assume that variation in female promiscuity simply mirrors variation in mating opportunities, as indicated by breeding density or synchrony [10, 11]. However, reviews of the empirical evidence have concluded that neither of these population traits can explain among-species variation in female promiscuity [5, 7, 12], though they may explain some variation at the intraspecific level [1, 8, 9], or within some restricted clades [13]. A plausible reason for their general failure at the broader scale is that female promiscuity is not a typical probabilistic behaviour; there are intrinsic species differences in the tendency of females to actively solicit, accept or reject copulations with multiple males [14–17]. For example, even colonial birds, where females normally have rich access to fertile males, can be strictly monogamous [12].

Alternative hypotheses have pinpointed a number of socio-ecological factors like social mating system [18, 19], kinship [20, 21], male parental care [22], sexual selection [8], life history variation [23], tropical versus temperate breeding [24] and seasonal migration [25]. Two of these stand out as having gained more support; promiscuous species typically have reduced levels of male parental care [7, 26–30] and fast life histories, i.e. high annual fecundity and short adult life span [7, 23]. However, whether such correlates can be regarded as causal factors is controversial. Major contrasts in male parental care and life history typically occur deep in the avian phylogeny, i.e. among taxonomic orders and families [8, 31, 32]. At these taxonomic levels, groups differ in many other aspects of ecological adaptations of possible relevance to promiscuity, which makes it difficult to identify the actual proximate and ultimate causes. Similarly, whether these broad correlates of female promiscuity apply within clades, where species are more similar in paternal care and life history strategies, but still variable in promiscuity, remains unclear.

Another challenge with comparative analyses of patterns of covariation is the possibility that the direction of causality can go either way. For example, the amount of male parental care may influence on the evolution of female promiscuity [22], but the level of male parental care can also be adjusted to the level of female promiscuity when males face a fitness trade-off between extrapair mating effort and parental effort [11]. Moreover, there might be no causal relationship at all when the studied variables are both intercorrelated with a true causal factor. Interpreting the results of comparative analyses of covariation among traits in terms of causal explanations can therefore be challenging and demands careful consideration of possible mechanisms.

The Passeriformes is the most speciose of all avian orders with ~ 6000 species. They display extensive ecological diversity, are found on all continents except Antarctica, and occur in nearly all terrestrial ecosystems [33]. Offspring are altricial and usually cared for by one or both parents, though there is considerable variation in the extent of paternal care [32, 33]. Passerines have faster life histories than the majority of the other avian orders, though their pace of life also varies considerably with body mass [34] and between tropical and temperate species [35]. Furthermore, passerines generally have higher rates of female promiscuity than other bird orders, but also the most variable rates [1, 12, 29]. This is especially true for members of the well-studied Passerides infraorder [36] (~ 3900 species), which includes species at both ends of the female promiscuity spectrum, that is, from strict sexual monogamy [37, 38] to most broods having mixed paternity [39, 40]. Here, we examine how male parental care, life history traits, and other hypothesized predictors (body size, sexual dichromatism, strength of pre-mating sexual selection, duration of social pair bonds, cooperative breeding, latitude and seasonal migration), covary with female promiscuity in a large data set of Passerides species. Our aim was to identify the most important correlates of female promiscuity and then critically evaluate whether these correlates can be regarded as causes or consequences, or the product of additional unknown causal factors.

## Methods

### Species and phylogeny

Our analysis included 202 species from 42 of the 67 families of Passerides (sensu Cracraft [36]). For the comparative analyses, we built a time-calibrated phylogeny using a supermatrix approach. Representative sequence data was downloaded for all species from the GenBank and BOLD repositories for three mitochondrial genes (cyt-b, ND2 and COI), three nuclear introns (Myo2, ODC and GAPDH), and one nuclear exon (RAG1) (see Additional file 1 for accession numbers). *Corvus corone* was used to root the tree. Individual genes were aligned using Muscle [41] in SeaView v4.5.4 [42] and we used Gblocks [43] to remove ambiguously aligned regions.

In order to obtain a time-calibrated phylogeny we analysed the concatenated dataset of all seven genes (6874 bp) in BEAST v1.8.4 [44]. We applied the best fitting model of nucleotide evolution to each gene partition as identified using the Bayesian Information Criterion in jModelTest2 [45]. We thus used GTR + I + Γ for cyt-b, ND2, COI, ODC and RAG1, HKY + Γ for Myo2, and K80 + Γ for GAPDH. Clock models were unlinked across all partitions and we applied a rate of 0.0145 substitutions per site per lineage (2.9%) per million years to the ND2 partition [46]. Relaxed uncorrelated lognormal distributions were used for the clock models and we assumed a Yule speciation process for the tree prior. We ran Markov Chain Monte Carlo chains for 50 million generations sampling every 5000 generation. We assessed convergence diagnostics using Tracer v1.6 [47], and we removed 25% of generations as burnin. Using TreeAnnotator and LogCombiner v1.8.4 [44], results were summarized as a posterior distribution of 1000 evenly sampled trees (Additional file 2) from which we also generated a maximum clade credibility tree using mean node heights.

### Female promiscuity

Female promiscuity was scored as an index equivalent to the proportion of extrapair young in a socially monandrous system. This index was derived from two sources of data. First, we compiled a comprehensive list of all socially monandrous Passerides species (*N* = 131; Additional file 3) for which the proportion of extrapair young could be extracted from published molecular paternity studies (*N* = 127), personal communication (*N* = 2) or our own unpublished data (*N* = 2). This list is twice as long as the one tabulated by Griffith et al. in 2002 [1], but is still strongly biased towards temperate zone species; very few paternity studies have been carried out in the tropics [48]. In cases of multiple paternity studies of the same species, we calculated the proportion of extrapair young from the added sample sizes. Second, we estimated the proportion of extrapair young from the coefficient of intermale variation in mean total sperm length (hereafter referred to as “sperm length CV”). This metric is strongly, negatively correlated with the proportion of extrapair young in passerine birds [49–51]. A similar relationship with sperm competition has also been documented in social insects [52] and rodents [53]. A common interpretation of this relationship is that sperm competition imposes stabilizing selection on sperm length such that it reduces the population variance around an optimal sperm length in proportion to the risk or intensity of sperm competition. We were able to estimate female promiscuity from sperm length CV for 129 species; of which 71 have no available data on extrapair young.

All sperm length measurements originated from sperm samples collected by our research groups over the past 12 years in Europe, North America, India, Australia and West Africa. Thus, they provide a considerable geographical and taxonomic addition to the paternity studies, and also include 32 species breeding in the tropics. In cases where we had sperm length data from multiple populations (or taxonomic subspecies) of the same species, we used the population with the highest number of sperm length measurements. Samples were obtained by cloacal massage [54] and fixed in formalin, and subsequently measured in a bright-field microscope with digital imaging software [55]. We calculated the mean length from 10 sperm cells per male. For each species, we calculated sperm length CV using the formula CV = (SD/X) × 100 × (1–1/4 N), where SD is the standard deviation of mean total sperm lengths, X is the population mean sperm length, and N is the number of males measured. This formula adjusts for the variation in sample size, since the coefficient of variation tends to be deflated at low sample sizes [56]. We only included species with a sample size of eight or more males (median = 15, maximum = 132). With a few exceptions (see Additional file 3), sperm samples are vouchered in the avian sperm collection at the Natural History Museum in Oslo (http://nhmo-birds.collectionexplorer.org).

**Fig. 1:**
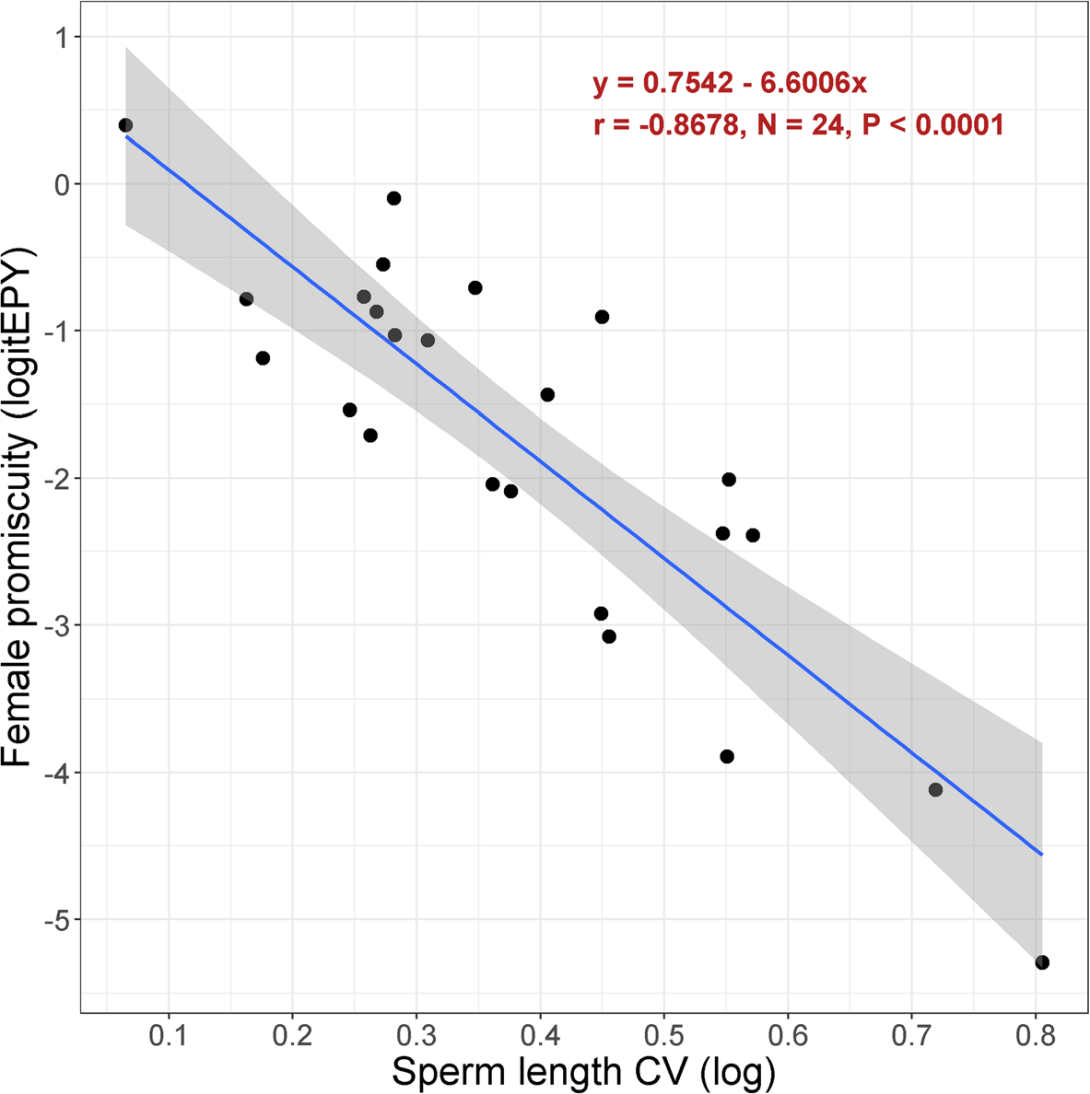
Relationship between the proportion of extrapair young in a population and the coefficient of total sperm length variation (sperm length CV) among males in the same population for 24 Passerides species (data in Additional file 4). The blue line is the linear regression line; its equation and the standardized regression coefficient are indicated. Shaded area show the 95% confidence interval for the regression line. The regression line was used to predict the proportion of extrapair young from estimates of sperm length CV in species without paternity data (see Additional file 3). Note that transformed values are used. A PGLS showed that the phylogenetic signal in this relationship (λ = 0.647) was not statistically different from λ = 0 (*P* = 0.126) and significantly lower than λ = 1 (*P* = 0.038; R^2^ = 0.774)

For the conversion of sperm length CV to the female promiscuity index, we refined the predictive fit of the regression model given in Lifjeld et al. [51] by selecting 24 species from the Passerides only, and from which we had paternity and sperm length data originating from the same study population (Additional file 4). Comparing data from the same study population is important to avoid noise in the model because extrapair paternity rates can vary geographically within a species [50, 57] and population variance in mean sperm lengths can vary accordingly [50]. Population mean sperm lengths can also change geographically [58–60], which implies that pooling individuals from different populations can inflate the variance estimate. We found that the sperm length CV explained 75% of the among-species variation in the proportion of extrapair young in the linear regression model (Fig. 1). The residual variance (25%) is probably due to sampling error in both variables (e.g. restricted sample sizes) and true temporal fluctuations in female promiscuity within populations [13, 61]. Presumably, the sperm length CV is a more stable population trait from year to year than extrapair paternity because sperm size has generally high heritability [62] and repeatability across seasons [63]. We used the regression line from this model to obtain a species-level estimate of the proportion of extrapair young for all 129 species with sperm length CV data (Fig. 1). A calculation sheet for converting sperm length CV into the estimate of proportion of extrapair young can be found in Additional file 3.

The two sources of data, paternity studies and sperm length CV, gave female promiscuity scores for a total of 202 species (Additional file 3). Fifty-eight species had scores derived from both sources. The two scores (logit-transformed) were positively correlated (Pearson r = 0.646, *N* = 58, *P* < 0.001). For these species we used the mean value of the two scores. The female promiscuity scores were logit-transformed prior to all statistical analyses.

### Predictor variables

We extracted scores on plumage colouration (“male colour”, “female colour” and “sexual dichromatism”) from the extensive data set published by Dale et al. [64]. In that study, plumage colouration was quantified in a single metric that expresses how “male-like” a particular plumage is. Briefly, for each sex in ~ 6000 passerine species (= ~ 12,000 data points), six plumage patches were scored in a three-dimensional (red, green and blue) colour space, and for each patch the percentage of the 120 (1%) nearest data points (in Euclidian space) being male, was scored. The mean score for the six patches was used as the colour score. The score therefore expresses how male-like a particular plumage is relative to other species, independently of the colour score of the other sex. The difference between the male and the female plumage score was used as the score for sexual dichromatism [64].

We also extracted scores from the five main predictor variables in the same study [64]: “body size”, “tropical life history”, “sexual selection”, “cooperative breeding” and “migration”. Three of these were compound variables; species with large “body size” had higher body mass and longer wings, those with a high score on “tropical life history” were more likely to breed in the tropics, inhabit areas of high environmental stability and lay smaller clutches, and those with more intense “sexual selection” tended to be socially polygynous, have more male-biased size dimorphism, and lack paternal care. “Cooperative breeding” was scored as present or absent, whereas migration was scored as no, partial or complete migration between breeding and non-breeding ranges. “Social bond” was taken from Tobias et al. [65]. This measure has three levels for the duration of social pair bonds: 1 = during courtship only, 2 = short-term (one breeding event), 3 = long-term (multiple breeding events).

In addition, we generated our own scores for “male parental care”, “migration distance”, “tropical/temperate” and “latitude”. For “male parental care”, we followed the approach of Arnold and Owens [7], where three temporal stages of parental care – nest building, incubation and chick feeding – were scored on an ordinal scale (0 = only female invests in parental care, 1 = both parents invests in care, but female invests more, 2 = male and female investment approximately equal, and 3 = only male invests). Data were taken from Handbook of the Birds of the World [33] and The Birds of North America Online [66]. The construction of “cock’s nests” – which are nests that are built in order to attract females – was not defined as male parental care. Incubation feeding (feeding the incubating female) was considered equivalent to a score of “1” in male incubation.

“Migration distance” was estimated as the linear distance between the study population and the centre of the winter distribution and rounded to the nearest 10^3^ km [33, 66, 67]. Migration distance for partial migrants and Afrotropical species was set to 0 assuming that the majority of the individuals migrate less than 500 km [67].

For “tropical/temperate”, tropical species were defined by the latitude of the study population(s); those within the tropical zone were classified as tropical, those outside as temperate. We also scored the “latitude” of the study population of each species, and calculated a mean latitude for multiple studies.

Species scores for all predictor variables used in the analyses are listed in Additional file 3.

### Statistical analyses

All statistical analyses were performed in R v. 3.4.1 [68]. For the comparative analyses we adopted a phylogenetic generalized least squares (PGLS) approach, using the *pgls* function in the *caper* package [69]. PGLS fits a linear regression of one or more predictor variables on a response variable in a phylogenetic framework, which takes into account the non-independent data points among related species [70]. This approach uses maximum likelihood to simultaneously optimize the phylogenetic signal (Pagel’s λ) in the residuals. We also estimated the phylogenetic signal in the response variable (female promiscuity) without entering any predictor variables in the model. We tested the effects of single predictor variables on the female promiscuity index by running the PGLS with the maximum clade credibility tree. We also tested for a curvilinear relationship by running a polynomial regression with the square of the predictor variable included. The analyses were also run with the 1000 sampled phylogenetic trees (Additional file 2) to evaluate the importance of phylogenetic uncertainty, and the results were qualitatively similar (Additional file 5).

We also ran a multivariate PGLS analysis, where we conducted a model selection approach to find the best model. We started out with including all predictor variables for all species with no missing values, and sequentially deleting the predictor with the highest *P*-value one by one until all remaining predictors were statistically significant (*P* < 0.05). All resulting models were compared with the Akaike Information Criterion (AIC). Among the models with the lowest AIC scores and within the range of 2 delta AIC scores [71, 72], we selected the most parsimonious one, i.e. with the fewest predictor variables, as the best model. We then reran this model with a maximized data set, including all species with no missing values for the included predictor variables.

Figures were generated in the R-package *ggplot2* [73] using species means without phylogenetic corrections.

## Results

**Fig. 2:**
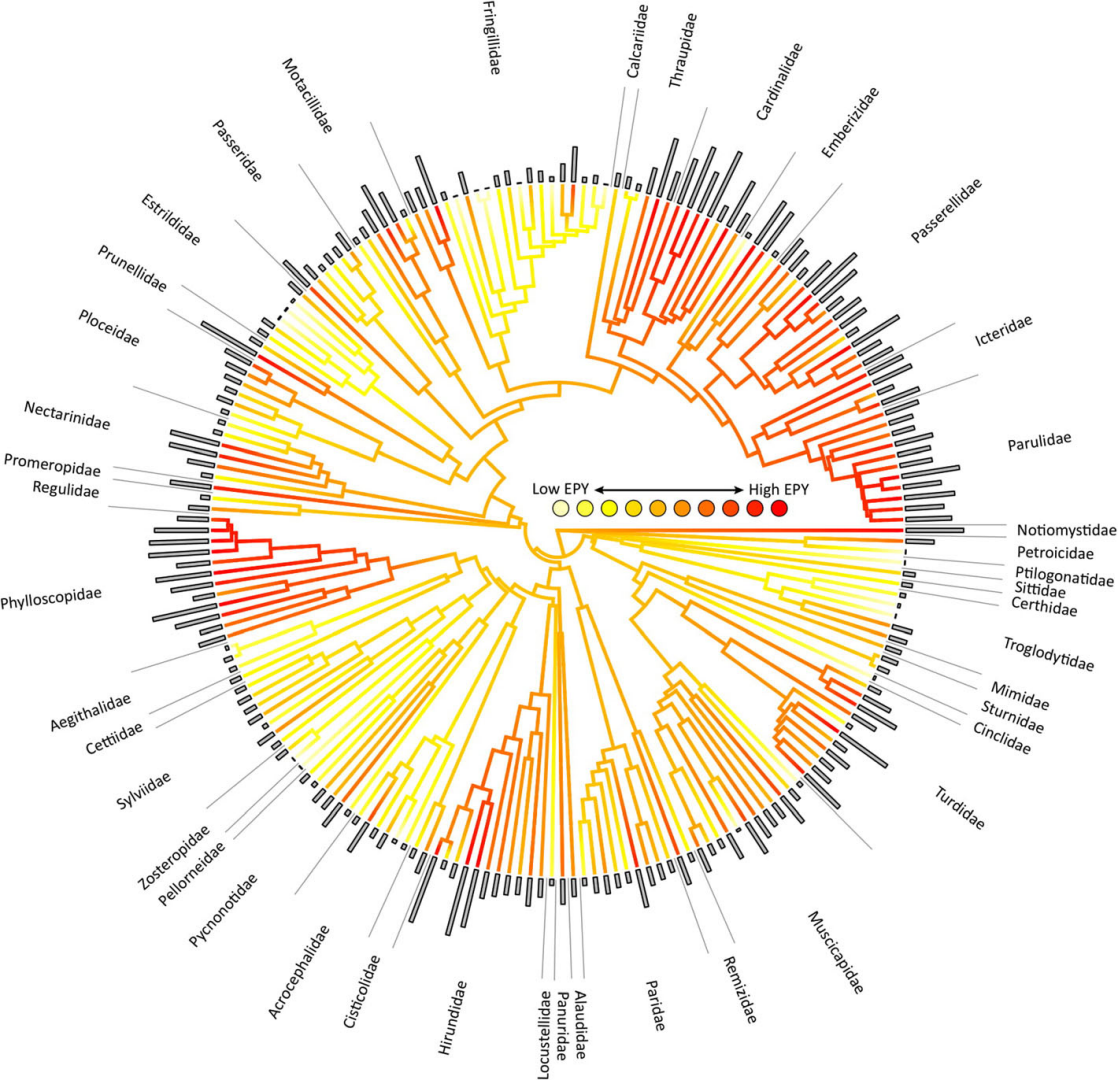
Phylogenetic distribution of female promiscuity estimates for the 202 Passerides species. A maximum clade credibility tree, derived from 1 000 trees, is shown. Female promiscuity estimates were derived from two sources: molecular paternity studies and the coefficient of total sperm length variation (for further details see Methods). Bars at tips indicate female promiscuity estimates for each species, with branch colouring indicating ancestral estimates of female promiscuity as inferred using the *contMap* function in the R package *phytools*. For better visualization (because of highly skewed data), species were binned into ten promiscuity categories ranging from 1 to 10, with 20–21 species in each category

Across the 202 species, female promiscuity scores varied from 0 (no promiscuity) to 0.580 (a majority of young being sired extrapair in a monandrous social situation), with a mean value of 0.180. This corresponds well to the mean percentage of 15.6% extrapair young reported in 65 Passerides species by Griffith et al. [1]. We note, that each species in our analyses is represented by a single estimate of promiscuity. While we acknowledge that there is temporal and geographic variation in female promiscuity within species, such intraspecific variation is challenging to incorporate into comparative analyses when not all species are represented by multiple estimates. Furthermore, generally speaking, intraspecific variation in female promiscuity is much lower than the variation among species [74]. The variation in female promiscuity across the Passerides phylogeny is visualized in Fig. 2. Families such as Hirundinidae, Muscicapidae, Turdidae, Fringillidae, Nectariniidae and Emberizidae showed particularly high variation in promiscuity among species, while other families had more consistently low (e.g. Estrildidae, Sylviidae, Pycnonotidae) or high (e.g. Phylloscopidae, Parulidae) levels of promiscuity. The phylogenetic signal in female promiscuity was moderate, as indicated by Pagel’s λ = 0.766; significantly lower than 1 (*P* < 0.001) and significantly higher than 0 (*P* < 0.001).

**Fig. 3:**
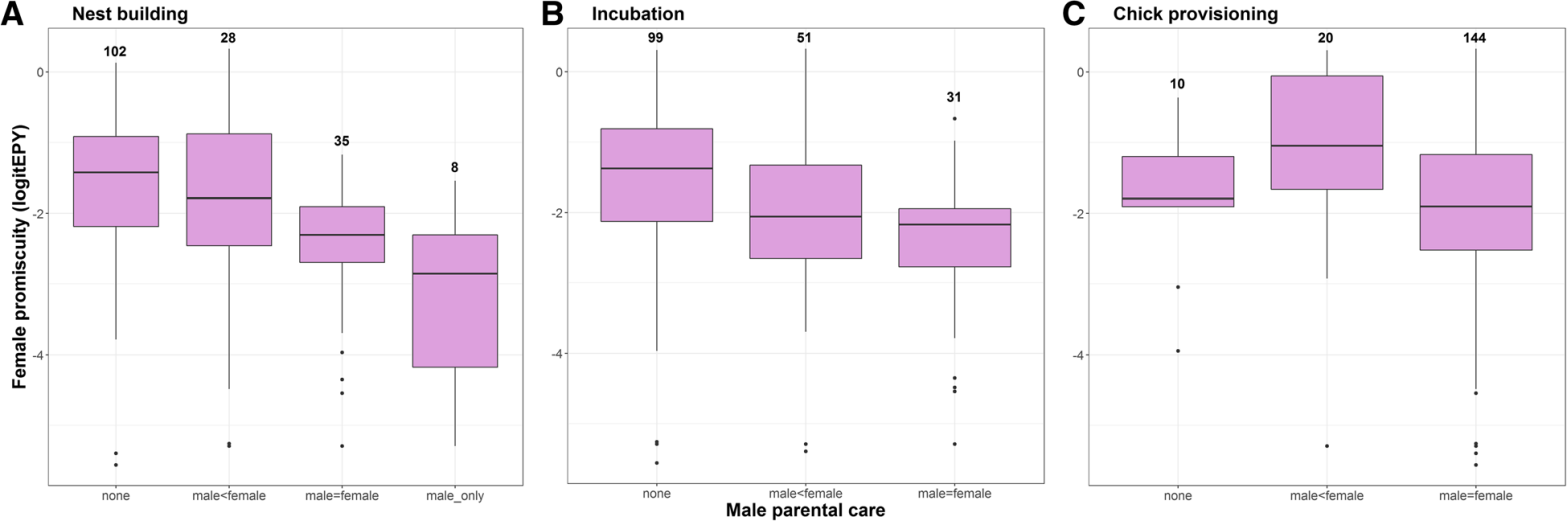
Boxplots showing the relationship between male parental care and female promiscuity. The pink boxes indicate the first and third quartiles where the internal line is the median; bars are 1.5 interquartile ranges with outliers indicated. Numbers indicate sample sizes. Plots are shown separately for the three stages of the breeding cycle: **a** nest building, **b** incubation, and **c** chick provisioning. Male parental care was scored as one of four categories: no male care, male care lower than female care, male care equal to female care, and male care only

**Fig. 4:**
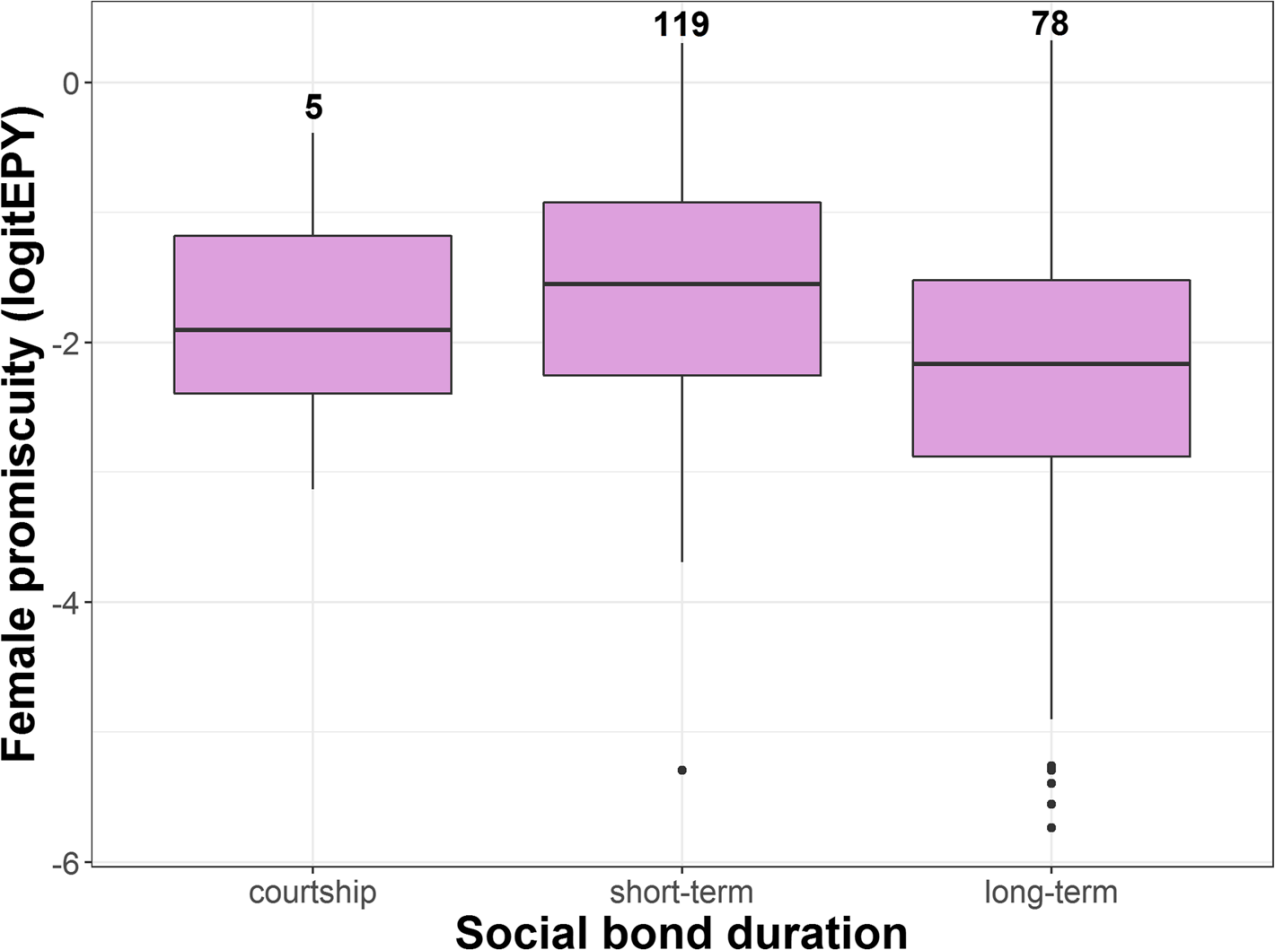
Boxplot showing the relationship between the duration of social pair bonds and female promiscuity. The pink boxes indicate the first and third quartiles where the internal line is the median; bars are 1.5 interquartile ranges with outliers indicated. Numbers indicate sample sizes. Data on social bond duration was taken from Tobias et al. [65]

**Fig. 5:**
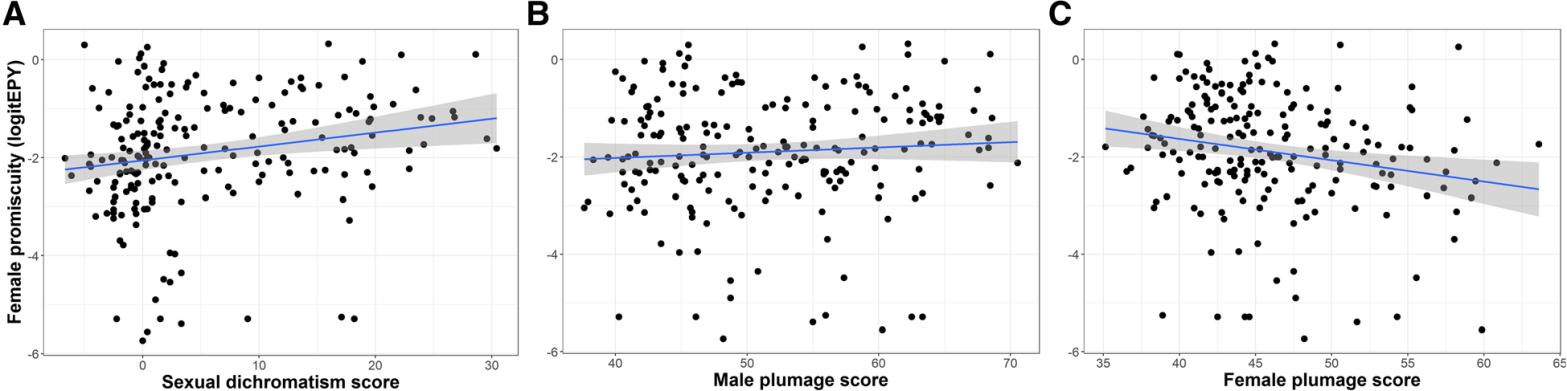
Scatterplots of the relationship between female promiscuity and measures of plumage colouration. **a** sexual dichromatism, **b** male plumage colouration, **c** female plumage colouration. Linear regression lines with 95% confidence intervals are indicated

**Fig. 6:**
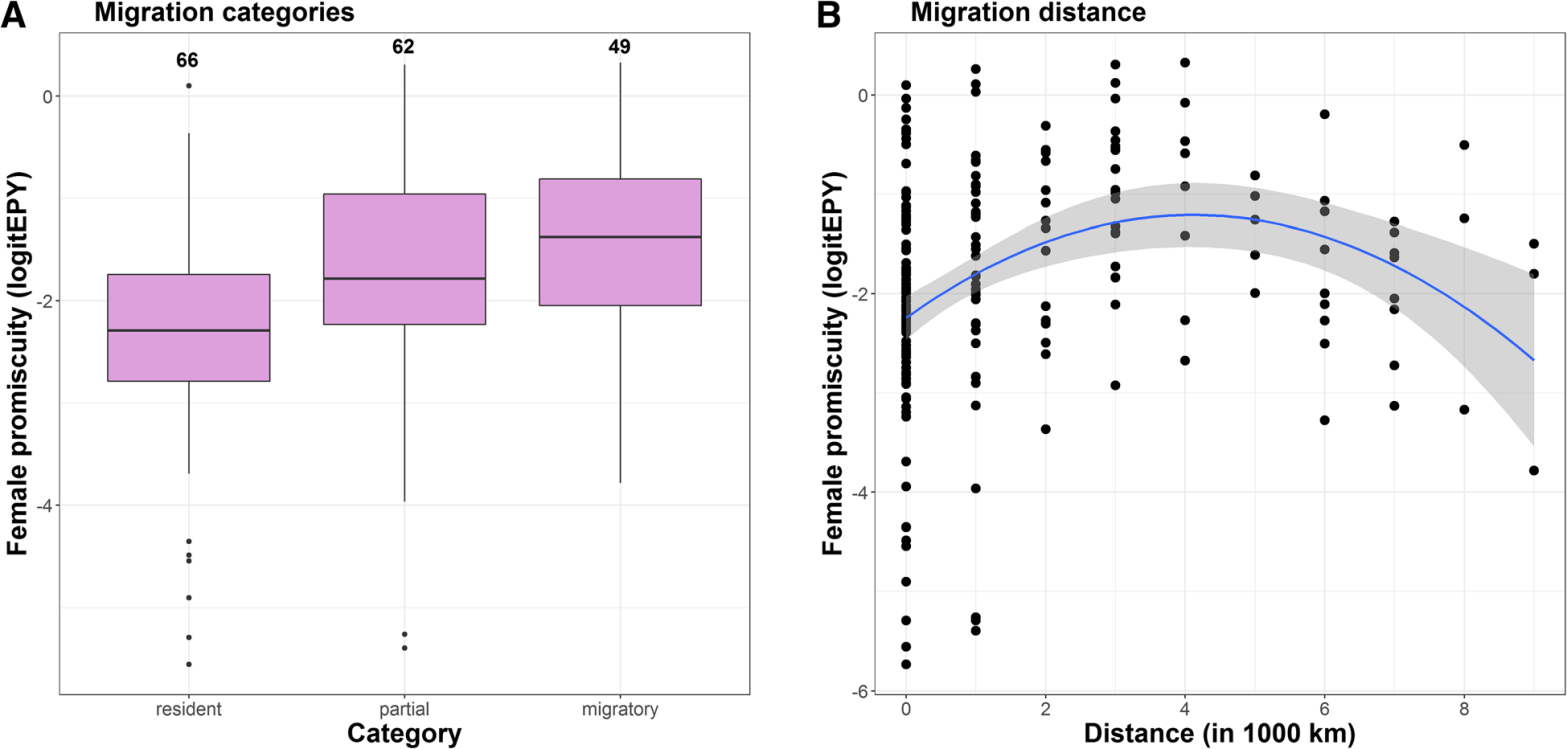
Relationship between migratory behaviour and female promiscuity. **a** Boxplot showing female promiscuity estimates for residents, partial migrants and full migrants. The pink boxes indicate the first and third quartiles where the internal line is the median; bars are 1.5 interquartile ranges with outliers indicated. Numbers are sample sizes. **b** Scatterplot showing female promiscuity as a function of migration distance. A polynomial regression line with 95% confidence interval is indicated for illustration

Five of the ten predictor variables were significantly associated with female promiscuity in bivariate PGLS analyses, though none of them explained more than 8.1% (*R*^2^) of the variation in female promiscuity (Table 1). First, as expected, there was a negative relationship with male parental care. However, the relationship was significant only at the nest building and incubation stage, not during chick provisioning (Table 1, Fig. 3). Second, species with long-term social pair bonds were significantly less promiscuous than those with short-term social bonds (Table 1, Fig. 4). Third, we found that more promiscuous species were more sexually dichromatic (Table 1, Fig. 5). When we analysed each sex separately, there was no effect of male colouration, but the effect of female colouration was significant (Table 1, Fig. 5); species in which females were duller were generally more promiscuous. Fourth, there was a significant effect of seasonal migration. Migratory species had more promiscuity than partial migrants and residents (Table 1, Fig. 6a). When these categories were further broken down into distance categories, it was evident that the relationship was curvilinear as shown in a polynomial regression (Table 1); promiscuity increased from residents and short-distance migrants to the medium-range migrants, and then decreased for the long-distance migrants (Fig. 6b). Finally, there was a significant positive effect of pre-mating sexual selection (Table 1), which indicates that female promiscuity increased with social polygyny, sexual size dimorphism and female-only parental care.

**Table 1:** PGLS results of single predictor variables on female promiscuity in Passerides songbirds

Predictor variable	N	Estimate (SE)	*t* (P)	*R* ^2^	λ
Male parental care					
Nest building	173	− 0.399 (0.099)	− 4.030 (**< 0.001**)	0.081	0.594
Incubation	181	−0.308 (0.122)	−2.525 (**0.012**)	0.029	0.497
Chick feeding	174	−0.239 (0.157)	−1.524 (0.129)	0.013	0.617
Plumage colouration					
Sexual dichromatism^a^	200	0.030 (0.010)	2.835 (**0.005**)	0.039	0.751
Male colour^a^	200	0.014 (0.011)	1.296 (0.197)	0.008	0.784
Female colour^a^	200	−0.036 (0.015)	−2.359 (**0.019**)	0.027	0.694
Migratory behaviour					
Categories^a^	177	0.235 (0.079)	2.988 (**0.003**)	0.049	0.604
Distance	202	0.326 (0.109)	3.006 (**0.003**)		
(Distance)^2^	202	−0.040 (0.015)	−2.729 (**0.007**)	0.044	0.711
Body size^a^	177	−0.037 (0.155)	−0.237 (0.813)	0.000	0.694
Tropical life history^a^	177	−0.176 (0.092)	−1.916 (0.057)	0.021	0.649
Latitude	202	0.001 (0.005)	0.264 (0.792)	0.000	0.761
Tropical/temperate	202	0.267 (0.216)	1.235 (0.218)	0.008	0.734
Social bond^b^	202	−0.368 (0.151)	−2.441 (**0.016**)	0.029	0.723
Sexual selection^a^	177	0.236 (0.098)	2.408 (**0.019**)	0.017	0.655
Cooperative breeding^a^	177	−0.016 (0.074)	−0.211 (0.833)	0.000	0.691

^a^Data from Dale et al. [64]. ^b^ Data from Tobias et al. [65]. Significant *P*-values are indicated in bold. All λ-values were significantly different (*P* < 0.001) from λ = 0 and λ = 1. The results using the maximum clade credibility tree (Fig. 2) are shown. For results based on the 1000 phylogenetic trees, see Additional file 5

The five non-significant predictors were body size, cooperative breeding, tropical life history, tropical versus temperate breeding, and latitude (Table 1), though tropical life history was close to statistical significance (*P* = 0.057; Table 1).

A possibility, however, is that the significant predictor variables are intercorrelated and partly explain the same variation in female promiscuity. Therefore, we also tested all predictor variables in a multivariate model. Here, the most parsimonious model, after a model selection approach (Additional file 6), included male parental care (nest building), social bond duration, sexual dichromatism and migration distance (Table 2). These factors all had significant partial effects, except for migration distance which only approached statistical significance in the maximized data set. It is worth noting that pre-mating sexual selection, which had a significant univariate effect (Table 1) was not included in the multivariate model (Table 2). The multivariate model explained 16.3% (adjusted R^2^) of the total variance in female promiscuity (Table 2).

**Table 2:** Results of the best multivariate PGLS model of female promiscuity in Passerides songbirds

Predictor	Estimate (SE)	t (P)
Intercept	−1.257 (0.498)	−2.523 (**0.013**)
Male nest building	−0.339 (0.097)	−3.504 (**0.001**)
Sexual dichromatism^a^	0.030 (0.010)	2.854 (**0.005**)
Migration distance	0.217 (0.118)	1.835 (0.068)
(Migration distance)^2^	−0.029 (0.015)	−1.896 (0.059)
Social bond^b^	−0.353 (0.165)	−2.135 (**0.034**)

Whole model: *F*_5,167_ = 7.703, *N* = 173, *P* < 0.001, adjusted *R*^2^ = 0.163, λ = 0.475

^a^Data from Dale et al. [64]. ^b^ Data from Tobias et al. [65]. The best model was chosen among all models ran from a reduced data set of 154 species with no missing values for any variable in a backwards stepwise deletion approach until only significant (*P* < 0.05) predictors remained. The model with fewest predictor variables was chosen from the set of best performing models (separated from the rest by delta AIC < 2; Additional file 6). The model shown here is based on a maximized data set (no missing values among the included predictor variables). The λ-value was significantly different from λ = 0 (*P* < 0.001) and λ = 1 (*P* < 0.001). Significant variables in bold (*P* < 0.05)

## Discussion

Our analyses identified four main variables that were significantly associated with variation in female promiscuity among Passerides songbirds: male parental care, duration of social pair bonds, sexual dichromatism and migration distance. In contrast, body size, tropical life history, latitude, tropical versus temperate breeding, pre-mating sexual selection, and cooperative breeding seemed to have little or no explanatory power. These findings partly confirm and partly contradict previous results of similar comparative approaches, usually based on smaller, but taxonomically more diverse data sets. This underscores the need for a hierarchical approach to dissect variance components in female promiscuity at different levels of the avian phylogeny [7, 8]. It is also necessary to critically evaluate the causal role of these factors, because of the correlative nature of the comparative approach.

### Male parental care

We found a significant negative association between female promiscuity and male parental care, a relationship that has also been documented in other, taxonomically broader, comparative studies [7, 8, 26–30]. Thus, an overall negative association seems robust. Bennett and Owens [8] analysed male parental care separately for the different phases of the nesting cycle, and found that female promiscuity was inversely related to male participation in nest building and incubation, but not to male share of nestling provisioning. We adopted the same methodology and found the same result with a much larger data set. Schwagmeyer et al. [30] also found that female promiscuity was elevated in species where males did not participate in incubation, while there was no relationship with male post-hatching care. In contrast, Møller and Birkhead [26] reported a significant association between female promiscuity and male post-hatching care, though Schwagmeyer et al. [30] questioned their analysis. A subsequent review of male parental care and paternity in birds [75] found no evidence for an interspecific relationship between paternity and male provisioning rates. Hence, there seems to be a general concordance among studies in that the interspecific association between male care and female promiscuity is restricted to the early stages of breeding, i.e. before hatching.

Theories predict causal relationships between female promiscuity and male parental care, but the direction of causality can go either way. As parental care has a fitness cost to males [76], males might be expected to withhold parental care in response to reduced paternity in their broods [77, 78]. This threat of retaliation might prevent females from being promiscuous, especially when male care is essential for female reproductive success. This is known as the “constrained female hypothesis” [79]. It predicts that female promiscuity should be inversely related primarily to post-hatch male care, because male chick provisioning is more essential for female reproductive success [27] and incurs higher mortality costs to males than the type of male care provided at the earlier stages [76]. The alternative scenario is that males adjust their pattern of parental care to the level of female promiscuity. This theory assumes a trade-off between male mating effort (extrapair activities) and male parental effort [11]. The trade-off will be shifted towards more mating effort, and consequently less parental effort, in species where females are more promiscuous, and especially early in the breeding season when more females are fertile [11, 80]. Hence, this “male trade-off hypothesis” predicts that the inverse relationship between male care and female promiscuity should be more pronounced during the early stages of the breeding cycle, which is consistent with previous evidence and our findings here. It also does not require any assumption of male retaliation in response to female promiscuity, which is theoretically questionable [78]. It therefore seems unlikely that male parental care is an evolutionary driver of female promiscuity in our study system. Instead, the variation in male parental care patterns among songbirds might be a consequence of the variation in female promiscuity.

### Pair bonds

Species with short-term pair bonds were more promiscuous than species with long-term pair bonds. This pattern is consistent with the “constrained female hypothesis” [79], which assumes that females reduce promiscuity because of the risk of male retaliation in the form of divorce. An alternative interpretation is that long-term pair bonds do not form so easily in species with high female promiscuity; because males spend relatively more reproductive effort on courting and attracting more females in such species, and hence less effort on pair bond maintenance and parental care, cf. “the male trade-off hypothesis” [11, 80]. These interpretations are therefore very parallel to those concerning male parental care. Our documentation of a negative relationship between female promiscuity and the duration of pair bonds is in close agreement with a previous comparative study that found a positive association between divorce and extrapair paternity in birds [81].

### Plumage dichromatism

Several comparative studies have documented a positive association between female promiscuity and sexual dichromatism [74, 82–84]. Owens and Hartley [83] showed that dichromatic species also had strongly sex-biased parental care. The correlation between female promiscuity and plumage dichromatism could therefore be a by-product of the correlation between female promiscuity and male parental care. However, when Owens and Hartley controlled for this bias statistically, the correlation between female promiscuity and dichromatism was upheld, which suggests an independent relationship. Our results from the multivariate analysis agree with this view. Dunn et al. [85] analysed a much bigger data set and found only borderline support for a relationship between sexual dichromatism and female promiscuity. Most studies have scored dichromatism as a difference between the two sexes [74, 83, 85], which precludes the opportunity to test for sex-specific associations. Møller and Birkhead [81] analysed each sex separately, and found that males became brighter with female promiscuity, while female colouration was unchanged. However, all studies have interpreted the association between sexual dichromatism and female promiscuity in support of sexual selection for brighter males.

Our results provide an interesting contrast to this traditional view. While we also found an association between sexual dichromatism and female promiscuity, the change occurred predominantly in females which were duller (less male-like) in more promiscuous species. We found no support for any relationship with male colouration. Our interpretation is that less male-like plumage could be a female adaptation to a promiscuous behaviour, in which crypsis might be advantageous, especially in species where females make extra-territorial forays [16, 86], and social signaling and social competition is less important for females [64]. Importantly, this idea reverses the causality, and considers female promiscuity as a cause, not an effect of sexual dichromatism.

### Migratory behaviour

Spottiswoode and Møller [25] reported that female promiscuity was positively associated with migration distance in birds, even when potential confounding factors such as latitude and breeding synchrony were controlled for statistically. A positive relationship between migration distance and female promiscuity was also detected by Pitcher et al. [87] and Gohli et al. [88], and our results corroborate these findings. Spottiswoode and Møller [25] listed several possible hypotheses for why long-distant migrants should be more promiscuous than resident species, but were hesitant to infer any causality because migration distance covaries with a number of other ecological variables (see also [87]). We suggest that pathogens can be important selection agents in this context. While all organisms need to fight off parasites and disease, migratory species must cope with several parasite communities during the annual cycle, whereas resident species only have to deal with one. Moreover, in the temperate region resident passerines are more adapted to plant food (seeds and berries) while migratory species feed more exclusively on insects and other invertebrates that are often vectors for endoparasites. In line with this reasoning, it is interesting to note that the two families with the highest and most stable levels of female promiscuity across species are the Old World leaf warblers (Phylloscopidae) and the New World wood warblers (Parulidae; Fig. 2), both of which are strictly insectivorous. Comparative studies have documented that migratory birds have higher parasite richness than their resident relatives [89, 90]. Migratory birds also have larger immune organs, i.e. bursa and spleen, which is consistent with the idea that they have to cope with a broader spectrum of parasites [91]. It is therefore a possibility that migration distance reflects a gradient in pathogen-mediated selection, which could select for more female promiscuity through a mechanism of female preference for compatible immune genes that enhance the survival of the offspring [92, 93]. Consistent with this idea is also the pattern that species with more promiscuity have larger spleens for their body size [83] and that in certain Passerides species offspring immune responsiveness is enhanced through female promiscuity [94–97]. Our finding that promiscuity was reduced for long-distance migrants (Fig. 6b) could possibly be explained by lower pathogen-mediated selection in species wintering in the southern temperate region, as opposed to those wintering in more tropical areas with a higher parasite diversity [98]. We encourage more comparative studies of pathogen loads and female promiscuity, especially within species with significant population differentiation in migratory behaviour.

### Life history

In the bivariate analyses the relationship between female promiscuity and tropical life history was close to statistical significance (Table 1), but the effect disappeared when other variables were taken into account (Table 2). Furthermore, there was no relationship with body size, which can be regarded as an additional proxy for pace of life in passerines [34], nor was there any relationship with latitude, or any difference in female promiscuity between tropical and temperate species. The lack of association with tropical breeding and life history variation is consistent with a previous comparative study that found no difference in female promiscuity between tropical and temperate passerines [67], but stands in contrast to work showing that female promiscuity was associated with fast life histories across the avian phylogeny [7, 8, 23]. The latter pattern has been considered consistent with the theory that reduction in male parental care in response to paternity loss may not be adaptive for short-lived species [77, 78], and thus that female promiscuity should be less constrained in short-lived species [7, 8, 23]. The fact that Passerides species display high variation in female promiscuity despite having a fairly restricted variation in life history traits, as compared to birds at large, argues against life history as a main driver for the evolution of female promiscuity. The hypothesis is also built on an assumption of male retaliation, that males withhold parental care in response to female promiscuity, which is not well supported by empirical data [75]. We therefore suggest that the association between life history and female promiscuity that exists for birds at large, is more likely a result of a third, unknown variable correlated with life history.

### Social mating systems

The composite variable for sexual selection, which reflects sexual size dimorphism, social polygyny and a lack of male care [64], showed a positive correlation with female promiscuity (Table 1), but the effect disappeared when we controlled for other variables, including male care (Table 2). As such, our results do not provide much support for a link between premating sexual selection and female promiscuity in Passerides songbirds. In a large comparative analysis, Pitcher et al. [87] found that social mating system was a strong predictor of relative testis mass in birds, as socially monogamous species had particularly small testes compared to polygynous ones. Relative testis size is frequently considered a proxy for female promiscuity [99, 100], but it is also plausible that the risk of sperm depletion drives the evolution of larger testes in polygynous species [87, 100]. There is in fact evidence from passerine birds that extrapair paternity is negatively related to the frequency of socially polygynous males in the population [18], so there is conflicting evidence as to how female promiscuity varies with social mating system.

We also found no association with cooperative breeding, though we note that our study is probably a weak test of the association, since cooperative breeding was scored as ‘absent’ or ‘present’. For many species there is only anecdotal evidence for the occurrence of cooperative breeding; rather few Passerides species are obligate cooperative breeders [19]. The empirical evidence for low promiscuity levels among cooperative breeders largely stems from other infraorders of the Passeriformes, especially Corvides and Meliphagides [19]. Thus, there seems to be no strong association between social mating systems and female promiscuity in Passerides songbirds. The only exception here is social polyandry, where females are socially paired with more than one male and hence copulate with more than one male as the norm [101, 102].

## Conclusion

Despite the large data set and the broad range of socioecological predictor variables analysed in our study, much of the variation in female promiscuity was left unexplained. Moreover, the causal effect of significant variables on female promiscuity, such as male parental care and sexual dichromatism, remains questionable. We argue that they are more likely responses to female promiscuity. In sum, the large variation in female promiscuity among Passerides songbirds remains an unresolved puzzle. Some recent studies have raised the idea that female promiscuity may not be adaptive [4, 6]. While this could be the case in some, perhaps primarily low-promiscuity species, we do not see how this idea can explain the high interspecific variability in the trait. With the assumption that female promiscuity entails some costs [4], there must be some adaptive value to this female reproductive strategy in species where it occurs at high frequency. Our recommendation is to intensify the search for factors responsible for a fitness benefit to females in highly promiscuous species, and test for their variable effects and covariation with female promiscuity rates across species. One promising candidate is pathogen-mediated selection which could vary in strength among related species with similar ecology, among populations of the same species with different ecologies, and within a lineage over time, because of the Red Queen co-evolutionary cycles between hosts and pathogens [103, 104]. Females may seek compatible immune genes through promiscuity, and more so when selection from pathogens is strong [92]. Indeed, this mechanism could be a possible explanation for the observed association between female promiscuity and migration distance in the present study, if migrants are under stronger natural selection from pathogens than are residents.

## Additional files

Additional file 1: Accession numbers for all species used to create the phylogenetic tree. All sequences downloaded from the GenBank (http://www.ncbi.nlm.nih.gov/genbank) or BOLD (http://www.boldsystems.org) database. In two instances (marked in yellow) sequences were lacking from the target species, and we used surrogate sequences from a closely related species. (XLSX 26 kb)

Additional file 2: NEXUS file of the 1000 phylogenetic trees used for analysis in this paper. (TXT 5863 kb)

Additional file 3: Data set for 202 species used in the comparative analyses. The second worksheet lists the references for paternity studies. The third sheet calculates the sperm length CV and the proportion of EPY from total sperm length data. The fourth sheet gives accession numbers in the Avian Sperm Collection in the Natural History Museum in Oslo, and total sperm length for all sperm samples used in the calculation of sperm length CV, except for two species: *Troglodytes aedon* (Becky Cramer, pers. comm.) and *Notiomystis cincta.* (Helen Taylor, pers. comm.). For samples from India (*Horornis fortipes*, *Larvivora brunnea*, *Passer rutilans* and *Phylloscopus occipitalis*) accession numbers refer to digital photos, as no samples were exported. (XLSX 170 kb)

Additional file 4: Data set of 24 species with sperm length CV and proportion of EPY from the same study population. These data were used to calculate the linear regression in Fig. 1. (DOCX 18 kb)

Additional file 5: Results of bivariate PGLS analyses with 1000 selected phylogenetic trees. (DOCX 17 kb)

Additional file 6: The model selection approach, using AIC, to find the best multivariate PGLS model as presented in Table 2. (XLSX 13 kb)

## Data Availability

The datasets supporting the conclusions of this article are included within the article and its additional supporting files. Sperm samples are deposited in the Avian Sperm Collection at Natural History Museum, Oslo (see Additional file 3). A database of the museum’s bird collections can be accessed online [http://nhmo-birds.collectionexplorer.org].
