# Supplementary material for: Evolution of female promiscuity in Passerides songbirds
Source: BMC Evol Biol. 2019 Aug 14;19:169. doi: 10.1186/s12862-019-1493-1 (PMC6694576; doi:10.1186/s12862-019-1493-1)
Supplement: Supplementary file 4 — Data set of 24 species with sperm length CV and proportion of EPY from the same study population. These data were used to calculate the linear regression in Fig. 1. (DOCX 18 kb) [file 12862_2019_1493_MOESM4_ESM.docx]

Additional File 4. Data on sperm length CV and the proportion of extrapair young from the same study population. These data were to calculate the linear regression equation in Figure 1.

| **Species** | **Locality** | **Sperm CV** | **Prop. EPY** |
| --- | --- | --- | --- |
| Acanthis flammea | Øvre Heimdalen, Norway | 2.299 | 0.115^1^ |
| Agelaius phoeniceus | QUBS, Ontario, Canada | 2.038 | 0.256^2^ |
| Carpodacus erythrinus | Czech Republic | 1.762 | 0.177^3^ |
| Cinclus cinclus | SE Norway | 5.240 | 0.016^4^ |
| Cyanistes caeruleus | Oslo, Norway | 2.378 | 0.110^5,6^ |
| Cyanistes teneriffae | Tenerife, Canary Island | 1.832 | 0.153^7^ |
| Delichon urbicum | Øvre Heimdalen, Norway | 2.545 | 0.192^8^ |
| Emberiza schoeniclus | Øvre Heimdalen, Norway | 1.852 | 0.295^9^ |
| Ficedula hypoleuca | Oslo, Norway | 2.856 | 0.044^10^ |
| Hirundo rustica | QUBS, Ontario, Canada | 2.818 | 0.288^11^ |
| Loxia curvirostra | SE Norway | 6.390 | 0.000^12^ |
| Luscinia svecica | Øvre Heimdalen, Norway | 1.916 | 0.263^13^ |
| Melospiza melodia | QUBS, Ontario, Canada | 2.812 | 0.051^14^ |
| Notiomystis cincta | Tiritiri Matangi, New Zealand | 1.162^15^ | 0.598^16^ |
| Parus major | Oslo, Norway | 3.526 | 0.085^5^ |
| Periparus ater | Lingen, Germany | 1.810^17^ | 0.317^18^ |
| Phoenicurus phoenicurus | SE Norway | 3.554 | 0.020^19^ |
| Phylloscopus trochilus | Øvre Heimdalen, Norway | 2.225 | 0.330^20^ |
| Poecile atricapillus | QUBS, Ontario, Canada | 3.567 | 0.118^21,22^ |
| Setophaga aestiva | QUBS, Ontario, Canada | 1.876 | 0.366^23^ |
| Setophaga ruticilla | QUBS, Ontario, Canada | 1.500 | 0.234^24^ |
| Sialia sialis | QUBS, Ontario, Canada | 3.727 | 0.084^25^ |
| Tachycineta bicolor | QUBS, Ontario, Canada | 1.913 | 0.475^26,27^ |
| Vermivora chrysoptera | QUBS, Ontario, Canada | 1.454 | 0.313^28^ |

**References**

1. Gunnhild Marthinsen & Jan T. Lifjeld, unpublished data

2. Weatherhead PJ, Boag PT. Pair and extra-pair mating success relative to male quality in red-winged blackbirds. Behav Ecol Sociobiol 1995, 37:81-91.

3. Albrecht T, Scnitzer J, Kreisinger J, Exnerová A, Bryja J, Munclinger P. Extra-pair paternity and the opportunity for sexual selection in long-distant migratory passerines. Behav Ecol 2007, 18:477-486.

4. Øigarden T, Borge T, Lifjeld JT. Extrapair paternity and genetic diversity: the white-throated dipper *Cinclus cinclus*. J Avian Biol 2010, 41:248-257.

5. Johannessen LE, Slagsvold T, Hansen BT, Lifjeld JT. Manipulation of male quality in wild tits: effects on paternity loss. Behav Ecol 2005, 16:747-754.

6. Krokene C, Lifjeld JT. Variation in the frequency of extra-pair paternity in birds: a comparison of an island and a mainland population of Blue Tits. Behaviour 2000, 137:1317-1330.

7. Garcia-Del-Rey E, Kleven O, Lifjeld JT. Extrapair paternity in insular African Blue Tits *Cyanistes teneriffae* is no less frequent than in continental Eurasian Blue Tits *Cyanistes caeruleus*. Ibis 2012, 154:862-867.

8. Whittingham LA, Lifjeld JT. High paternal investment in unrelated young: extra-pair paternity and male parental care in house martins. Behav Ecol Sociobiol 1995, 37:103-108.

9. Kleven O, Lifjeld JT. No evidence for increased offspring heterozygosity from extrapair mating in the reed bunting (*Emberiza schoeniclus*). Behav Ecol 2005, 16:561-565.

10. Lifjeld JT, Slagsvold T, Lampe HM. Low frequency of extra-pair paternity in pied flycatchers revealed by DNA fingerprinting. Behav Ecol Sociobiol 1991, 29:95-101.

11. Kleven O, Jacobsen F, Robertson RJ, Lifjeld JT. Extrapair mating between relatives in the barn swallow: a role for kin selection? Biol Lett 2005, 1:389-392.

12. Kleven O, Bjerke B-A, Lifjeld JT. Genetic monogamy in the common crossbill (*Loxia curvirostra*). J Ornithol 2008, 149:651-654.

13. Johnsen A, Lifjeld JT. Ecological constraints on extra-pair paternity in the bluethroat. *Oecologia* 2003, 136:476-483.

14. Beth MacDougall-Shackleton, personal communication.

15. Helen A. Taylor, personal communication.

16. Brekke P, Wang J, Bennett PM, Cassey P, Dawson DA, Horsburgh GJ, Ewen JG. Postcopulatory mechanisms of inbreeding avoidance in the island endemic hihi (*Notiomystis cincta*). Behav Ecol 2012, 23:278-284.

17. Schmoll T, Kleven O. Sperm dimensions differ between two Coal Tit *Periparus ater* populations. J Ornithol 2011, 152:515-520.

18. Schmoll T, Dietrich V, Winkel W, Epplen JT, Schurr F, Lubjuhn T. Paternal genetic effects on offspring fitness are context dependent within the extrapair mating system of a socially monogamous passerine. *Evolution* 2005, 59:645-657.

19. Kleven O, Øigarden T, Foyn B, Moksnes A, Røskaft E, Rudolfsen G, Stokke B, Lifjeld J. Low frequency of extrapair paternity in the common redstart (*Phoenicurus phoenicurus* ). *J Ornithol* 2007, 148:373-378.

20. Bjørnstad G, Lifjeld JT. High frequency of extra-pair paternity in a dense and synchronous population of willow warblers *Phylloscopus trochilus*. J Avian Biol 1997, 28:319-324.

21. Mennill DJ, Ramsay SM, Boag PT, Ratcliffe LM. Patterns of extrapair mating in relation to male dominance status and female nest placement in black-capped chickadees. Behav Ecol 2004, 15:757-765.

22. Otter K, Ratcliffe L, Michaud D, Boag PT. Do female black-capped chickadees prefer high ranking males as extra-pair partners? Behav Ecol Sociobiol 1998, 43:25-36.

23. Yezerinac SM, Weatherhead PJ, Boag PT: Extra-Pair Paternity and the Opportunity for Sexual Selection in a Socially Monogamous Bird (*Dendroica petechia*). Behav Ecol Sociobiol 1995, 37:179-188.

24. Reudink MW, Marra PP, Kyser TK, Boag PT, Langin KM, Ratcliffe LM. Non-breeding season events influence sexual selection in a long-distance migratory bird. Proc R Soc B 2009, 276:1619-1626.

25. Meek SB, Robertson RJ, Boag PT. Extrapair paternity and intraspecific brood parasitism in eastern bluebirds revealed by DNA fingerprinting. Auk 1994, 111:739-744.

26. Delmore KE, Kleven O, Laskemoen T, Crowe SA, Lifjeld JT, Robertson RJ. Sex allocation and parental quality in tree swallows. Behav Ecol 2008, 19:1243-1249.

27. Stapleton MK, Kleven O, Lifjeld JT, Robertson RJ: Female tree swallows (*Tachycineta bicolor*) increase offspring heterozygosity through extrapair mating. Behav Ecol Sociobiol 2007, 61:1725-1733.

28. Vallender R, Friesen V, Robertson R. Paternity and performance of golden-winged warblers (*Vermivora chrysoptera*) and golden-winged X blue-winged warbler (*V. pinus*) hybrids at the leading edge of a hybrid zone. Behav Ecol Sociobiol 2007, 61:1797-1807.
