# Supplementary material for: Evolution of female promiscuity in Passerides songbirds
Source: BMC Evol Biol. 2019 Aug 14;19:169. doi: 10.1186/s12862-019-1493-1 (PMC6694576; doi:10.1186/s12862-019-1493-1)
Supplement: Supplementary file 5 — Results of bivariate PGLS analyses with 1000 selected phylogenetic trees. (DOCX 17 kb) [file 12862_2019_1493_MOESM5_ESM.docx]

Additional File 5. PGLS results of single predictor variables on the female promiscuity index accounting for phylogenetic uncertainty. Means and 95% confidence intervals are given for the bivariate models run with 1000 phylogenetic trees.

| **Predictor** | **N** | **Estimate** | **P** | **R^2^** | **Lambda** |
| --- | --- | --- | --- | --- | --- |
| Male parental care |  |  |  |  |  |
| Nest building | 173 | -0.398 (-0.405, -0.391) | <0.001 (<0.0001, 0.0001) | 0.086 (0.083, 0.089) | 0.502 (0.480, 0.525) |
| Incubation | 181 | -0.308 (-0.318, -0.298) | 0.012 (0.010, 0.016) | 0.034 (0.032, 0.037) | 0.457 (0.231, 0.532) |
| Chick feeding | 174 | -0.239 (-0.248, -0.231) | 0.129 (0.116, 0.141) | 0.013 (0.013, 0.014) | 0.585 (0.363, 0.653) |
| Plumage colouration |  |  |  |  |  |
| Sexual dichromatism | 200 | 0.030 (0.029, 0.031) | 0.005 (0.004, 0.006) | 0.039 (0.037, 0.042) | 0.732 (0.689, 0.779) |
| Male colour | 200 | 0.015 (0.014, 0.015) | 0.195 (0.166, 0.224) | 0.008 (0.007, 0.010) | 0.775 (0.740, 0.811) |
| Female colour | 200 | -0.036 (-0.037, -0.035) | 0.020 (0.015, 0.025) | 0.027 (0.025, 0.029) | 0.681 (0.635, 0.724) |
| Migratory behaviour |  |  |  |  |  |
| Categories* | 177 | 0.235 (0.230, 0.240) | 0.003 (0.003, 0.004) | 0.048 (0.046, 0.051) | 0.602 (0.553, 0.652) |
| Distance | 202 | 0.326 (0.319, 0.333) | 0.003 (0.002, 0.005) |  |  |
| Distance^2^ | 202 | -0.040 (-0.040, -0.039) | 0.007 (0.006, 0.008) | 0.044 (0.042, 0.046) | 0.700 (0.645, 0.737) |
| Body size* | 177 | -0.036 (-0.046, -0.027) | 0.816 (0.769, 0.864) | 0.000 (0.000, 0.000) | 0.690 (0.642, 0.724) |
| Tropical life history* | 177 | -0.176 (-0.181, -0.171) | 0.056 (0.050, 0.064) | 0.021 (0.019, 0.022) | 0.648 (0.601, 0.695) |
| Latitude | 202 | 0.001 (0.001, 0.002) | 0.786 (0.728, 0.848) | 0.000 (0.000, 0.001) | 0.752 (0.714, 0.787) |
| Tropical/temperate | 202 | 0.269 (0.251, 0.287) | 0.215 (0.187, 0.246) | 0.008 (0.007, 0.009) | 0.721 (0.680, 0.763) |
| Social bond** | 202 | -0.367 (-0.378, -0.356) | 0.016 (0.013, 0.019) | 0.029 (0.027, 0.030) | 0.715 (0-678, 0.752) |
| Sexual selection* | 177 | 0.236 (0.231, 0.241) | 0.017 (0.015, 0.019) | 0.032 (0.031 0.034) | 0.652 (0.605, 0.699) |
| Cooperative breeding* | 177 | -0.016 (-0.020,- 0.012) | 0.827 (0.786, 0.871) | 0.000 (0.000, 0.000) | 0.688 (0.642, 0.734) |

*Data from Dale et al. [64]. **Data from Tobias et al. [65].
